# Recruiting adults of ethnic minorities into clinical trials: a synthesis of strategies

**DOI:** 10.12968/bjca.2023.0032

**Published:** 2023-09-01

**Authors:** Nirmala Sam, James Hill, Oliver Hamer

**Affiliations:** 1 Lancashire Teaching Hospitals NHS Foundation Trust, Lancashire, UK; 2 University of Central Lancashire, Preston, UK; 3 National Institute for Health and Care Research Applied Research Collaboration − Northwest Coast, UK

**Keywords:** Systematic review, Renal, Recruitment, Ethnic minorities, Nursing

## Abstract

There is a long history of BAME under-representation in medical research. Underrepresentation of minority ethnic groups have been assessed by several studies, showing that black and minority ethnic groups were less likely to participate and engage in medical research when compared to white British groups (in relation to education, occupation, health, belief, and attitudes to medical research).There may be several strategies that improve inclusivity, including translation of participant information, culturally specific recruitment, and adaptations to the invitation process. However, with a dearth of literature in the area, there is now a need to contextualise these strategies in relation to renal research.

## Introduction

There is a long history of black, Asian and minority ethnic (BAME) under-representation in UK research (Mccarthy 1994; Smart and Harrison 2017). Underrepresentation of minority ethnic groups have been assessed by several studies, showing that black and minority ethnic groups were less likely to participate and engage in medical research (in relation to education, occupation, health, belief, and attitudes to medical research) (Cronin and Ward 2004; Smart and Harrison 2017). There are several factors to the underrepresentation of BAME participants such as language difficulties, stigma, lack of research awareness, mistrust of research, and general inaccessibility to locations whereby research is conducted (George et al. 2013; Smart and Harrison 2017; Teo et al. 2017; Waheed et al. 2015). That said, there is little tailoring for the recruitment of BAME participants by researchers who design and conduct studies (Wright 2020). Researchers suggests that a lack of translation services, added time to the recruitment process, and insufficient training are some of the key barriers they encounter when recruiting ethnic minorities (Haley et al. 2017). This issue is particularly problematic in renal research whereby participants of BAME ethnicity are greatly underrepresented (Ahmed et al. 2021). There is need for research to address this given that black and south Asian communities have higher rates of chronic kidney disease compared to their white counterparts (Gardiner et al. 2021).

The challenge of addressing the under-representation of BAME individuals in medical research is multifaceted, requiring significant time and effort, including the need to consider societal and cultural factors (Gardiner et al. 2021; Wright 2020). However, given the disproportionate impact of renal failure on BAME populations, there is an immediate need for the government, researchers, and healthcare professionals to prioritize inclusivity in research studies. In recent years, research has highlighted several strategies to improve inclusivity within research trials (Masood et al. 2019). However, with a dearth of literature in the area, there is now a need to contextualise these strategies in relation to renal research. This commentary aims to critically appraise the methods used within the review by Masood et al, 2019 and expand upon the findings in the context of clinical practice (Masood et al. 2019).

## Methods

The systematic review conducted a comprehensive search strategy including four databases: Medline, Embase, PsychInfo and CINAHL. In addition, authors also performed manual search of the reference list of each included study. The review included randomized controlled trials and participants who were all non-white adults from the United Kingdom (18 years or above). Any health-related intervention was eligible for inclusion. The primary outcomes were descriptions of recruitment processes and descriptions of recruitment procedures. The review excluded studies with mixed populations recruitment, non RCTs, review articles, qualitative studies, cost analysis reports, conference reports, and studies excluding ethnic minorities from recruitment (Masood et al. 2019).

Two reviewers independently screened the titles, abstracts and full texts of publications against the selection criteria. A third author resolved any disagreements. Data synthesis was carried out by two reviewers by grouping various recruitment strategies into broader themes. The quality of the studies included was not assessed within the review. This was because trials did report the effectiveness of the strategies on recruiting rates (Masood et al. 2019).

## Results

After removing duplicates, a total of 2309 studies were identified. Following full text and reference screening, a total of 21 eligible trials were included. All 21 studies included ethnic minorities in clinical trials within the UK. Of the 21 trials, seven were focused on diabetes, six on mental health, two on heart diseases, two on smoking, one on multimorbid conditions, one on post-natal support, one on kidney disease and one on asthma. Studies were based in both primary and secondary care. A thematic synthesis identified key recruitment strategies employed by the studies to encourage ethnic minority participants to clinical trials (within the UK). Studies were grouped according to three main strategies for recruiting ethnic minority participants to UK clinical trials: (1) adaptation of screening and outcome measures; (2) culturally specific recruitment training; and (3) the recruitment process (Masood et al. 2019).

### Theme 1: adaptation of screening and outcome measures

A total of five studies adapted screening measures to include translation, and culturally adapted measured. Researchers in these studies used forward or backwards translation and employed both professionals and lay bilingual panels to assist in this process. Eight further studies adapted outcome measures with translation and offered explanations in other languages.

### Theme 2: Culturally specific recruitment training

A total of four studies provided culturally sensitive recruitment training for staff and encouraged the adoption of an interview schedule that was culturally sensitive to ethnic groups. One of the studies provided two weeks of culturally specific research training to their research staff, and another study encouraged research staff to recognise cultural differences and focus on communication skills with participants.

### Theme 3: Recruitment processes

#### Study invitation process

In total, two trials collaborated with religious settings, including mosques and faith leaders, to promote their projects, raise awareness of mental health, and engage with attendees. Additionally, eight trials worked with ethnic minority community organizations to encourage referrals, facilitate the engagement process between researchers and participants, and overcome mistrust. Some of these trials (n= 5) also utilized day centres that catered to high numbers of ethnic minority participants for recruitment. Two trials referred to recruiting family members and friends of already recruited participants. Almost all trials recruited from ethnically dense areas, except for two. Moreover, three trials provided transport provisions to facilitate the participation of South Asian women, and four trials arranged home visits and telephone follow-up calls to recruit ethnic minority participants. Overall, these strategies were implemented to increase inclusivity in research and enhance participation among underrepresented groups.

#### Patient information and engagement

In terms of engaging with participants who could not read English, two trials used multilingual invites and posters, while three trials translated patient information sheets into targeted ethnic languages. Two trials also provided audio recordings of patient information in addition to multilingual invitations. For British Asian recruitment, options for screening and interviewing were offered in hospital clinics, GP surgeries, or participants’ homes. Bilingual staff were used in two trials to explain study details to patients who were unable to read any language, while other trials utilized language interpreting services during the introduction of the trial and delivery of interventions. In addition, six trials offered participants the option to be interviewed in their preferred language of Urdu, Punjabi, or English. These strategies aimed to enhance inclusivity and engagement with participants from diverse ethnic backgrounds.

#### Awareness of cultural practises

To promote cultural sensitivity and inclusivity, 11 trials used ethnic matching by providing recruitment staff from the same cultural background, and some utilized ethnically matched link workers and translators. Additionally, four trials offered culturally appropriate foods and products to patients, while one trial took into consideration cultural and religious festive periods when planning recruitment, assessment, and evaluation sessions. These efforts aimed to create a more welcoming and accommodating environment for participants from diverse cultural backgrounds, and to address potential barriers to recruitment and retention in the trials.

### Commentary

This systematic review scored nine out of 11 on the JBI Checklist for Systematic Reviews and Research Syntheses (Aromataris et al. 2015). There were two unfilled criteria due to the lack of quality appraisal of included studies and an absence of discussion related to publication bias (Aromataris et al. 2015). The systematic review met its aims by employing a narrative synthesis to provide an overview of existing evidence (Masood et al. 2019). That said, the review cannot be judged to be of high quality because it did not assess the methodologically quality of included studies, and therefore the validity and reliability of the synthesis may be limited in its implications for practise.

The primary recruitment strategies used in clinical trials included adaptation of screening and outcome measures, culturally specific recruitment training and cultural awareness (with patient engagement) during the recruitment process (Masood et al. 2019). However, the lack of tailored approaches from researchers emerged as a major theme in the review. From the findings, it was evident that recruiting ethnic minorities requires consideration of cultural requirements, including various strategies that take into account cultural and linguistic needs, as well as the researchers’ awareness of cultural expectations (Masood et al. 2019).

There were several key strategies highlighted by the review to support the recruitment of ethnic minority participants into research (Masood et al. 2019). Initially, the review highlighted that research teams could ensure they involve bilingual and bicultural research staff to assist with the adaptation of screening materials that may be given to potential participants (Waheed et al. 2015). This strategy is likely to be more successful if research staff are viewed by ethnic minorities as culturally competent (insiders), and have the ability to explain in lay terms, western concepts of medicine (Chen et al. 2005). Further studies not included in this review, have recommended ways to strengthen this strategy with the employment of cultural consultants to ensure that recruiting terminology is appropriate, promoting inclusion and reducing avoidance (Le et al. 2008). A further strategy that supports recruitment of ethnic minorities is to ensure that researchers undergo training in culturally specific recruitment approaches (Masood et al. 2019). This training may last up to two weeks and include topics on avoiding stigmatisation (Loue and Sajatovic 2008), awareness of religion (e.g., congregational loyalty and traditions) (Areán et al. 2003), developing culturally appropriate interpersonal skills, understanding cultural correctness (e.g., women’s involvement with husband consent) (Miranda et al. 1996) and adoption of culturally sensitive language (e.g., avoiding culturally threatening wordings) (Le et al. 2008). The final key strategy highlighted by the review is to ensure research staff have a focus on cultural awareness and engagement during the recruitment process (Masood et al. 2019). Recruitment of ethnic minorities can be promoted by recruiting from ethnically diverse areas, community/religious organizations, using ethnic matching at sites, conducting interviews in preferred languages, providing telephone follow-up, and offering interpreting services.

In addition to the strategies outlined in the review, other studies have highlighted that the patient– physician relationship is a key factor in recruiting ethnic minorities (Anjorin and Lipsky 2018; Fry et al. 2021). Literature suggests that patients learn about clinical trials through their physician, and so sociocultural differences between the patient and the physician can exacerbate challenges to participation (e.g., understanding the study aims and the potential benefit/ harm) (Anjorin and Lipsky 2018). Overcoming these challenges with the encouragement of constructive relationships may lead to positive outcomes towards recruiting minority patients into clinical trials (Fry et al. 2021). Literature also suggests that that having a lead researcher from the same ethnic background as the participant may also improve ethnic minority participation in research (Fry et al. 2021). Employing this evidence, NHS trusts may want to consider ethnic matching when making clinic appointments, as having a range of staff with different sociocultural backgrounds could improve participation of minority patients into research (Fry et al. 2021). These initiatives should be integrated into ongoing trials, and participants should be randomly assigned to either the usual recruitment methods or the culturally sensitive recruitment methods (prior to commencement of trial randomisation or allocation of the trial). By randomly assigning participants to the two recruitment methods, we can evaluate the impact of culturally sensitive recruitment approaches.

From the current evidence, recommendations could be made to improve interpretation services within NHS trusts in the UK (Ali and Watson 2018). Trusts may also need research information/leaflets to be provided in the patient’s own language, hospitals to provide multilingual written information sheets, and have staff member who can speaks participant’s language (or understand cultural traditions relevant to the research) (Islam et al. 2015). The widespread introduction of the ‘NIHR-INCLUDE’ guidance provides a framework for trusts to encourage recruitment of ethnic minority patients into research (NIHR 2021). The guidance makes suggestions on questions to guide the deliberations of funders, researchers and delivery teams, as they design and assess health research proposals, providing examples of good practice and other resources to improve inclusion of under-served groups (NIHR 2021).

The current evidence suggests that there are gaps in the use and reporting of recruitment strategies for ethnic minorities in clinical trials (Masood et al. 2019). Future research should aim to develop a standard reporting guideline which supports authors to detail their approaches within clinical trials that recruit ethnic minorities. In addition, research teams should be encouraged to undergo culturally sensitive training prior to attaining ethical approval for studies so that they become increasing inclusive of ethnic minorities. Culturally sensitive training is particularly important to equip researchers with the necessary skills to recruit and retain these hard-to-reach groups (Masood et al. 2019). Future research should also evaluate the impact of recruitment strategies on patients with kidney disease as there is a dearth of literature in this area (Masood et al. 2019). Only one study was identified within the current review that included participants with kidney disease, and it was unknown if participants were on dialysis, underwent a transplant or were on conservative management (Masood et al. 2019). Further high-quality trials are needed in this area to assess the effectiveness of these strategies, including groups of researchers who have undergone culturally specific recruitment training.

## CPD Reflective Questions

- What are the main strengths and weaknesses of the systematic review?
- What barriers need to be overcome before implementing new strategies aimed at recruiting ethnic minority participants?
- What are the key facilitators that could improve the success of recruitment strategies for ethnic minorities into UK trials?

## Figures and Tables

**Table 1:** Critical appraisal of the systematic review using the JBI Checklist for Systematic Reviews and Research Syntheses.

*JBI items	Responses
1. Is the review question clearly and explicitly stated?	Yes, the review aimed to describe strategies for recruiting ethnic minority participants to UK clinical trials.
2. Were the inclusion criteria appropriate for the review question?	Yes, the review states that they are conducting a systematic review of published RCT’s to describe strategies for recruiting ethnic minority participants to UK clinical trials.
3. Was the search strategy appropriate?	Yes, search strategy included relevant terms for the search.
4. Were the sources and resources used to search for studies adequate?	Yes, a systematic literature search was conducted from four bibliographic databases in consultation with two systematic reviewers and a librarian specialising in systematic reviews by applying three terms.
5. Were the criteria for appraising studies appropriate?	Yes, two authors independently read and reviewed the titles and abstracts of trials and full texts were checked against the selection criteria. They also had discussions within the team to resolve uncertainties and disagreements.
6. Was critical appraisal conducted by two or more reviewers independently?	No, critical appraisal of included studies was not assessed.
7. Were there methods to minimize errors in data extraction?	Yes, three authors were involved in designing a data extraction sheet which was tested on first three papers. Data extraction of the remaining papers was also conducted by three other authors.
8. Were the methods used to combine studies appropriate?	Yes, a narrative synthesis was conducted as the review did not intend to combine studies with meta-analysis.
9. Was the likelihood of publication bias assessed?	No, the review did not explore publication bias.
10. Were recommendations for policy and/or practice supported by the reported data?	Yes, the review made recommendations for practise from the available data within the included studies.
11. Were the specific directives for new research appropriate?	Yes, the review makes clear recommendations for future research and identifies gaps in evidence.

## Key Points

- There are several strategies for recruiting ethnic minority participants to UK clinical trials which include ethnic matching and language translation.
- Recruiting ethnic minorities requires consideration of their cultural requirements, including strategies that take into account societal and linguistic needs.
- Further high-quality trials are needed in renal research to assess the effectiveness of strategies for recruiting ethnic minority participants.
